# Effects of Different Microplastics on Wheat’s (*Triticum aestivum* L.) Growth Characteristics and Rhizosphere Soil Environment

**DOI:** 10.3390/plants13243483

**Published:** 2024-12-12

**Authors:** Yan Zhang, Songze Hao, Ping Li, Zhenjie Du, Yuze Zhou, Guohao Wang, Zhijie Liang, Ming Dou

**Affiliations:** 1 Farmland Irrigation Research Institute, Chinese Academy of Agricultural Sciences, Xinxiang 453002, China; zhangyan09@caas.cn (Y.Z.); liping05@caas.cn (P.L.); 2 School of Water Conservancy and Transportation, Zhengzhou University, Zhengzhou 450001, China; zyzaaaab@gmail.com; 3 Henan Provincial Technical Center for Ecology and Environment, Zhengzhou 450046, China; ayshsz1@163.com (S.H.); xxxhao95@163.com (G.W.); 4 Laboratory of Quality and Safety Risk Assessment for Agro-Products on Water Environmental Factors, Ministry of Agriculture, Xinxiang 453002, China; imdzj11@163.com; 5 Agricultural Water Soil Environmental Field Research Station of Xinxiang, Chinese Academy of Agricultural Sciences, Xinxiang 453002, China

**Keywords:** microplastics (MPs), wheat, growth characteristics, physicochemical properties, enzymatic activities, rhizosphere soil

## Abstract

In order to reveal the effects of microplastics (MPs) on the growth and rhizosphere soil environmental effects of wheat (*Triticum aestivum* L.), three microplastic types (polypropylene MPs (PP-MPs), high-density polyethylene MPs (HDPE-MPs), and polylactic acid MPs (PLA-MPs)), particle sizes (150, 1000, and 4000 μm), and concentrations (0.1, 0.5, and 1 g·kg^−1^) were selected for a pot experiment under natural environment conditions. The differences in germination rate (*GR*), germination inhibition rate (*GIR*), growth characteristics, physicochemical properties, and enzymatic activities of wheat in rhizosphere soil were analyzed using statistical analysis and variance analysis. The results show that the germination rate of wheat seeds decreased under different MPs, and the HDPE-MPs, medium particle size (1000 μm), and medium concentration (0.5 g·kg^−1^) had the greatest inhibitory effect on wheat seed germination. The effects of MPs on wheat seed growth characteristics were inconsistent; the germination potential (*GP*), germination index (*GI*), and vitality index (*VI*) showed a significant decreasing trend under the PLA-MPs and medium-concentration (0.5 g·kg^−1^) treatment, while the mean germination time (*MGT*) showed a significant increasing trend; the *GP* and *MGT* showed a significant decreasing and increasing trend under the high-particle-size (4000 μm) treatment, respectively, while the *GI* and *VI* showed a significant decreasing trend under the medium-particle-size (1000 μm) treatment. The growth characteristics of wheat plants showed a significant decreasing trend under different MPs, with the SPAD, nitrogen concentration of the leaves, and plant height decreasing the most under PLA-MP treatment, the SPAD and nitrogen concentration of leaves decreasing the most under low-particle-size (150 μm) and low-concentration (0.1 g·kg^−1^) treatments, and the decreases in plant height under the high-particle-size (4000 μm) and high-concentration (1 g·kg^−1^) treatments being the largest. There were significant increasing trends for ammonium nitrogen (NH_4_^+^), total phosphorus (TP), soil urease (S-UE), soil acid phosphatase (S-ACP), and soil sucrase (S-SC) under different microplastics, while the PLA-MPs had a significant increasing trend for nitrate nitrogen (NO_3_^−^) and a significant decreasing trend for pH; there was a significant decreasing trend for total nitrogen (TN) under the HDPE-MPs and PLA-MPs, and for each particle size and concentration, the PLA-MPs and low-concentration (0.1 g·kg^−1^) treatments showed a significant decreasing trend for soil catalase (S-CAT). The research results could provide certain data and theoretical bases for evaluating the effects of MPs on crop growth and soil ecological environments.

## 1. Introduction

Microplastics (MPs), as a new pollutant, have the characteristics of relatively small volume, strong ability to adsorb pollutants, and large persistence and environmental harm [1], which has meant that MPs have become a globally recognized environmental problem, widely existing in soil ecological environment systems and bringing harm to farmland ecological environments and food security [2].

MPs in soil mainly include *in situ* MPs and exogenous input MPs [3]. *In situ* MPs mainly refer to the MPs produced by weathering and cracking plastic residues in the soil, and the plastic residues are mainly agricultural mulch residues in the agricultural production process. Exogenous input MPs refer to MPs that enter the soil environment from the outside world through processes such as atmospheric sedimentation, surface runoff, irrigation, sludge, and organic fertilizer application [4,5,6,7,8]. Irrigation is an important way for MPs to enter farmland soil. On the one hand, MPs are widely found in surface freshwater bodies (rivers and lakes) available for irrigation; on the other hand, sewage irrigation has important applications in many parts of the world, especially in arid and semi-arid areas, and there is a higher concentration of MPs in sewage, and after sewage irrigation, sewage sludge is additionally used in the form of fertilizer in agricultural fields [9,10]. For example, the use of mulching film and renewable water resources measure 1.342 million tons and 17.58 billion m^3^ in 2022, according to the China Rural Statistical Yearbook (2023) and China Water Resources Bulletin (2022), respectively, and they directly or indirectly increase the source of soil MPs; in order to avoid soil pollution caused by agricultural films, the People’s Republic of China introduced measures for the management of agricultural films in 2020.

At present, studies on the effects of MPs on plant growth have mainly involved wheat (*Triticum aestivum* L.) [11,12], corn (*Zea mays* L.) [13], soybean (*Glycine max* (L.)) [14], pakchoi (*Brassica campestris* L.) [15,16], water spinach (*Ipomoea aquatica Forsk*) [17], mung bean (*Vigna radiata* (L.)) [18], lettuce (*Lactuca sativa*) [19], peanut (*Arachis hypogaea*) [20], green broad bean (*Vicia faba*) [21], pea (*Pisum sativum* L.) [22], melon (*Cucumis melo* L.) [23], cucumber (*Cucumis sativus* L.) [24], onion (*Allium cepa*) [25], cresley (*Lepidium sativum*) [26], ryegrass (*Lolium perenne* L.) [27], etc. These studies mainly analyze the effects of different MPs (types, particle sizes, and concentrations) on the growth characteristics of different crops and vegetables through hydroponics and soil culture experiments, investigating the main germination rate (*GR*), germination inhibition rate (*GIR*), germination potential (*GP*), germination index (*GI*), vitality index (*VI*), mean germination time (*MGT*), plant height, antioxidant enzyme catalase (CAT), superoxide dismutase (SOD), peroxidase (POD), ascorbate peroxidase (APX), ascorbate oxidase (AAO), glutathione reduction enzyme (GR), leaf area, leaf fresh mass, root fresh mass, chlorophyll *a* (Chl-*a*), chlorophyll *b* (Chl-*b*), chlorophyll *a*+*b* (Chl-*a*+*b*) concentration, etc. Xiao et al. analyzed the emergence rate, plant height, whole plant biomass, and above-ground dry matter distribution of wheat under the addition of high-density polyethylene MPs (HDPE-MPs) [28]. Li et al. found that PVC-MPs’ single stress caused greater effects on soybean chloroplast PSII than PE-MPs’ single stress and combined stresses [29]. Chen et al. found that the polystyrene (PS-MPs) (5 μm) had no significant effects on the *MGT*, *GI*, and *VI* of wheat, corn, millet, and sunflower seeds, while peanuts were sensitive to PS-MPs (5 μm), and the related indices were significantly inhibited, the inhibition being stronger with a higher microplastic concentration [30]. On the whole, these studies showed that MPs all stressed the germination of crop seeds and had different effects on their growth characteristics.

In addition, relevant studies have shown that polypropylene (PP), PS, polyethylene (PE), polyvinyl chloride (PVC), low-density polyethylene (LDPE), HDPE, polylactic acid (PLA), poly (butylene adipate co-terephthalate) (PBAT), poly (methyl methacrylate) (PMMA), and phenol-formaldehyde (PF) MPs have certain effects on the physicochemical properties and enzyme activities in soil [31,32,33,34,35,36,37,38]; for example, Fei et al. found that the PE-MPs and PVC-MPs inhibited the FDAse activity in soil, and the FDAse activity decreased by 7% and 5%, respectively [34]. Wang et al. [36], Lu et al. [37], and Xin et al. [38] analyzed the effects of PBAT/PLA-MPs on the dissolved organic carbon (DOC), dissolved organic nitrogen (DON), enzyme activity, and physicochemical properties in soil, respectively. Relevant studies have shown that the PLA-MPs decreased pH value but increased the C/N ratio, and the medium concentration of PLA-MPs increased DOC concentration but slowed down the decline rate of ammonium nitrogen (NH_4_^+^); however, the effects of PE-MPs and PP-MPs on the physicochemical properties were significantly different, which was mainly due to the fact that PLA-MPs were easier to degrade and significantly affected the physicochemical properties in soil [39]. Ma et al. found that the PE-MPs reduced the organic matter (OM) by 33.9% [40], and Zhang et al. found that the OM decreased by 0.8% after the application of plastic film residue, indicating that the possible reason for the decline of the OM in soil was that the addition of MPs reduced the enzyme activity in soil [41]. Ren et al. found that the PE-MPs (5%) had no significant effect on the soluble organic carbon (SOC) in soil [42], and Wang et al. found that the FDAse activity decreased by 10.2% in residual white mulch soil [43].

The study of the effects of MPs on crop growth is still in its infancy. Wheat was selected as a test crop in this study, and different microplastic types, particle sizes, and concentrations were set to reveal the effects of different MP occurrence environments on wheat seed germination rate, growth characteristics, physicochemical properties, and enzyme activities of rhizosphere soil through pot experiments under natural environments, in order to provide data support and technology for studying the mechanism by which MPs influence crop growth and the ecological environment effect of rhizosphere soil.

## 2. Results and Analysis

### 2.1. Effects of MPs on Wheat Seed Germination Rate

**Figure 1:**
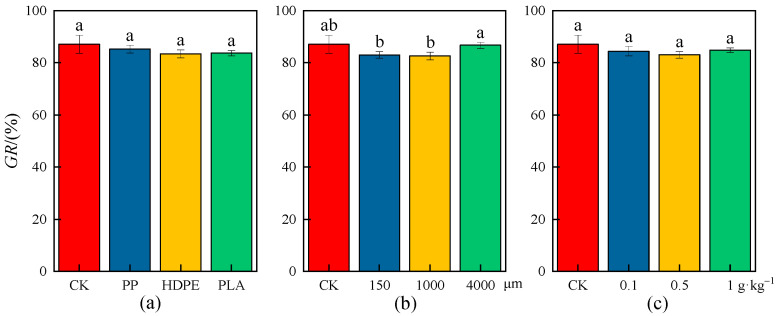
*GR* of wheat seeds under the CK and different microplastic characteristics. (**a**) Microplastic types. (**b**) Microplastic particle sizes. (**c**) Microplastic concentrations. All data are presented as the mean ± SD (n ≥ 3 biological replicates). Lowercase letters above bars indicate significant differences according to the post hoc Fisher LSD test (*p* < 0.05).

**Figure 2:**
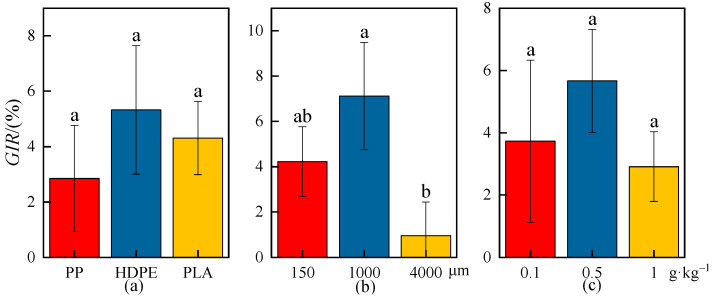
*GIR* of wheat seeds under the CK and different microplastic characteristics. (**a**) Microplastic types. (**b**) Microplastic particle sizes. (**c**) Microplastic concentrations. All data are presented as the mean ± SD (n ≥ 3 biological replicates). Lowercase letters above bars indicate significant differences according to the post hoc Fisher LSD test (*p* < 0.05).

The effects of MPs on the *GR* and *GIR* of wheat seeds are shown in Figure 1 and Figure 2, there were no significant differences in the *GR* and *GIR* of wheat seeds among different microplastic-type, particle-size, and concentration treatments (*p* < 0.05). The average *GR* of wheat seeds under different microplastic types, particle sizes, and concentrations was lower than that in the CK; for example, the average *GR* of wheat seeds was 87.11%, 85.28%, 83.46%, and 83.75% under the CK, PP-MP, HDPE-MP, and PLA-MP treatments, respectively, which indicated that the average germination level of wheat seeds was decreased to some extent under exposure to MPs.

The average *GIR* of wheat seeds was highest for the HDPE-MP treatment, followed by the PLA-MP treatment and then the PP-MP treatment, indicating that the HDPE-MPs had the greatest inhibitory effect on wheat germination, while the PP-MPs had the least inhibitory effect. The average *GR* of wheat seeds was the minimum, 82.67%, when the microplastic particle size was 1000 μm, and the average *GIR* was the maximum, 7.12%. The average *GR* of wheat seeds increased with low particle size (150 μm) and high particle size (4000 μm), and the average *GIR* was 0.96% when the particle size was 4000 μm. The average *GR* of wheat seeds was the minimum, 83.11%, when the microplastic concentration was 0.5 g·kg^−1^, and the average *GIR* was the maximum, 5.66%; the average *GR* of wheat seeds was increased and the average *GIR* was decreased when the microplastic concentrations were 0.1 g·kg^−1^ and 1 g·kg^−1^.

In general, the inhibitory effects on wheat seed germination were greatest when the MPs were medium-particle-size (1000 μm) and medium-concentration (0.5 g·kg^−1^), while the inhibitory effects on wheat seed germination were weakened when the MPs were low/high-particle-size and concentration, which may be caused by the properties of the MPs themselves or by the agglomeration of the MPs that reduces the accessibility of wheat to MPs.

### 2.2. Effects of MPs on Wheat Seed Growth Characteristics

**Figure 3:**
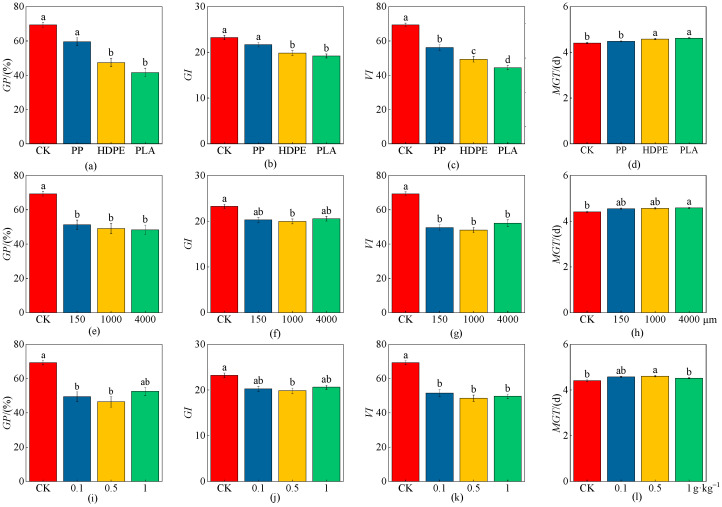
Effects on wheat seed growth (*GP*, *GI*, *VI*, and *MGT*) under the CK and different microplastic characteristics. (**a**–**d**) Microplastic types. (**e**–**h**) Microplastic particle sizes. (**i**–**l**) Microplastic concentrations. All data are presented as the mean ± SD (n ≥ 3 biological replicates). Lowercase letters above bars indicate significant differences according to the post hoc Fisher LSD test (*p* < 0.05).

As shown in Figure 3 and Table 1, the *GP* and *GI* under the HDPE-MP and PLA-MP treatments had significant decreasing trends compared with CK (*p* < 0.05), and the *VI* had a significant decreasing trend under different microplastic-type treatments (*p* < 0.05). The *MGT* was significantly increased under the HDPE-MP and PLA-MP treatments (*p* < 0.05); the decreases in the *GP*, *GI*, and *VI* were 66.55%, 21.00%, and 55.98% under the PLA-MP treatment, respectively, while the increase in *MGT* was 4.67% (Figure 3a–d). The *GP* and *VI* under different particle-size (150, 1000, and 4000 μm) treatments had significant decreasing trends compared with CK (*p* < 0.05), the *GI* had a significant decreasing trend under the medium particle size (1000 μm) (*p* < 0.05), and the *MGT* was significantly increased under the high particle size (4000 μm) (*p* < 0.05). The maximum reduction in the *GP* was 43.56% under the high particle size (4000 μm), the maximum reductions in the *GI* and *VI* were 16.52% and 43.99% under the medium particle size (1000 μm), respectively, and the maximum increase in *MGT* was 3.83% under the high particle size (4000 μm) (Figure 3e–h). The *GP* had significant decreasing trends under the low concentration (0.1 g·kg^−1^) and medium concentration (0.5 g·kg^−1^) compared with CK (*p* < 0.05), the *GI* and *MGT* had significant changing trends under the medium concentration (0.5 g·kg^−1^) (*p* < 0.05), and the *VI* had a significant decreasing trend under each concentration (0.1, 0.5, and 1 g·kg^−1^) (*p* < 0.05); the *GP*, *GI*, and *VI* decreased by 49.04%, 16.91%, and 42.63% under the medium concentration (0.5 g·kg^−1^), respectively, while the *MGT* increased by 4.23% (Figure 3i–l).

### 2.3. Effects of MPs on Growth Characteristics of Wheat Plants

**Figure 4:**
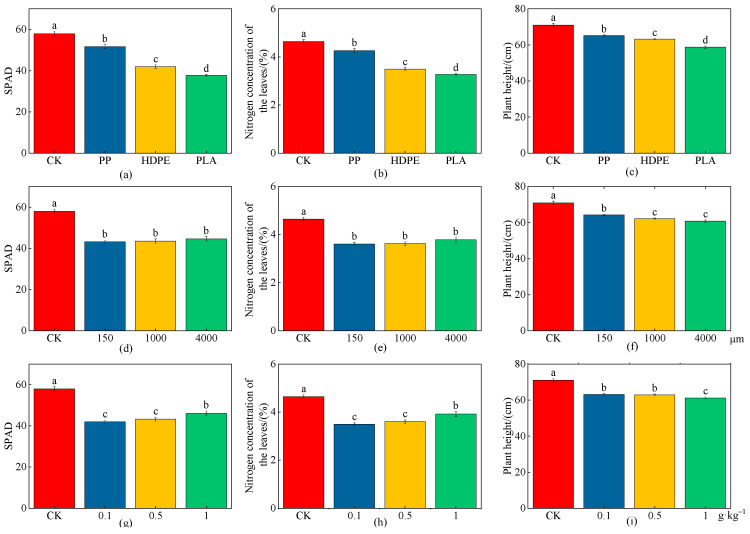
Effects on wheat plant growth (SPAD, nitrogen concentration of the leaves, and plant height) under the CK and different microplastic characteristics. (**a**–**c**) Microplastic types. (**d**–**f**) Microplastic particle sizes. (**g**–**i**) Microplastic concentrations. All data are presented as the mean ± SD (n ≥ 3 biological replicates). Lowercase letters above bars indicate significant differences according to the post hoc Fisher LSD test (*p* < 0.05).

As shown in Figure 4 and Table 2, the SPAD, nitrogen concentration of the leaves, and plant height under different microplastic types, particle sizes, and concentrations showed significant decreasing trends compared with CK (*p* < 0.05), indicating that different MPs had certain inhibitory effects on the SPAD, nitrogen concentration of the leaves, and plant height. The SPAD, nitrogen concentration of the leaves, and plant height had significant differences under different microplastic types (*p* < 0.05), and the maximum reductions in SPAD, nitrogen concentration of the leaves, and plant height were 53.74%, 41.91%, and 20.77% under the PLA-MPs, respectively (Figure 4a–c). The decreasing trends of SPAD and nitrogen concentration of the leaves were not significant under different microplastic particle sizes, and the maximum decreases in SPAD and nitrogen concentration of the leaves were 34.31% and 28.44% under the low particle size (150 μm); there were significant differences in plant height under different microplastic particle sizes (*p* < 0.05), and the maximum reduction in plant height was 16.77% under the high particle size (4000 μm) (Figure 4d–f). The SPAD, nitrogen concentration of the leaves, and plant height had significant differences under different microplastic concentrations (*p* < 0.05); the maximum reductions in SPAD and nitrogen concentration of the leaves were 38.04% and 32.89% under the low concentration (0.1 g·kg^−1^), respectively, while the maximum reduction in plant height was 16.04% under the high concentration (1 g·kg^−1^) (Figure 4g–i).

### 2.4. Effects of MPs on Physicochemical Properties of Wheat Rhizosphere Soil

**Figure 5:**
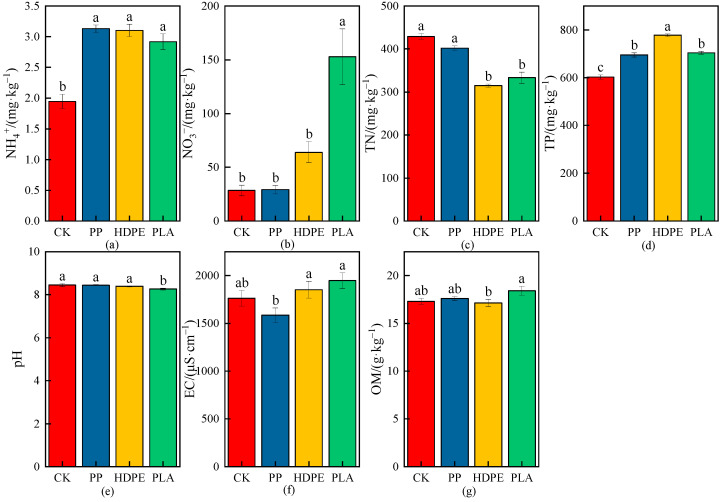
Effects on physicochemical properties of wheat rhizosphere soil under the CK and different microplastic types. (**a**) NH_4_^+^, (**b**) NO_3_^−^, (**c**) TN, (**d**) TP, (**e**) pH, (**f**) EC, and (**g**) OM. All data are presented as the mean ± SD (n ≥ 3 biological replicates). Lowercase letters above bars indicate significant differences according to the post hoc Fisher LSD test (*p* < 0.05).

**Figure 6:**
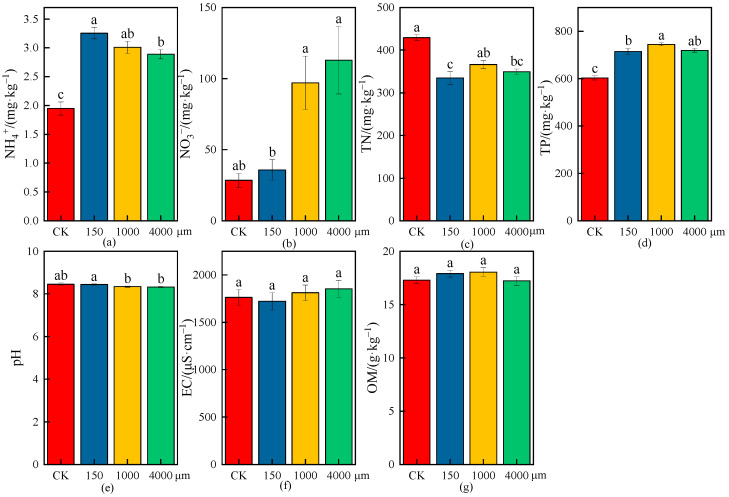
Effects on physicochemical properties of wheat rhizosphere soil under the CK and different microplastic particle sizes. (**a**) NH_4_^+^, (**b**) NO_3_^−^, (**c**) TN, (**d**) TP, (**e**) pH, (**f**) EC, and (**g**) OM. All data are presented as the mean ± SD (n ≥ 3 biological replicates). Lowercase letters above bars indicate significant differences according to the post hoc Fisher LSD test (*p* < 0.05).

**Figure 7:**
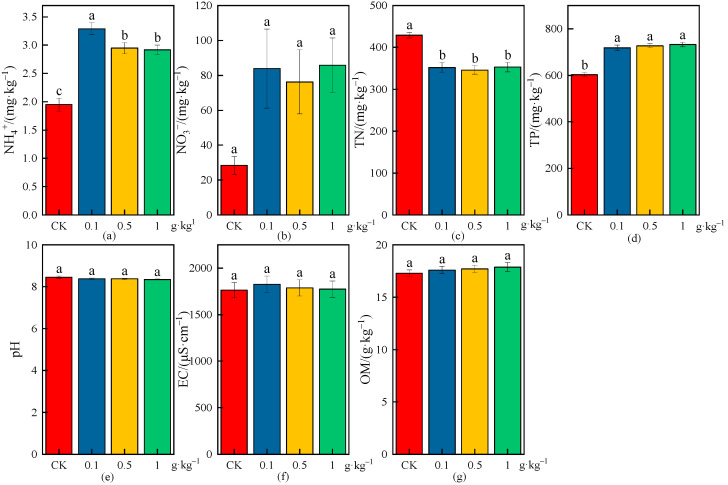
Effects on physicochemical properties of wheat rhizosphere soil under the CK and different microplastic concentrations. (**a**) NH_4_^+^, (**b**) NO_3_^−^, (**c**) TN, (**d**) TP, (**e**) pH, (**f**) EC, and (**g**) OM. All data are presented as the mean ± SD (n ≥ 3 biological replicates). Lowercase letters above bars indicate significant differences according to the post hoc Fisher LSD test (*p* < 0.05).

The effects of different MPs on physicochemical properties of wheat rhizosphere soil are shown in Figure 5, Figure 6 and Figure 7, and the increase or decrease ranges of the average values of physicochemical indices in rhizosphere soil under various treatments compared with CK are shown in Table 3.

As shown in Figure 5 and Table 3, the NH_4_^+^ concentrations had significant differences under different microplastic types compared with CK (*p* < 0.05), and the NH_4_^+^ concentrations increased by 60.63%, 59.26%, and 49.84% under the PP-MPs, HDPE-MPs, and PLA-MPs, respectively. There were significant differences in NH_4_^+^ concentrations between the microplastic types, and the NH_4_^+^ concentration increased the most under PP-MPs, indicating that PP-MPs had the greatest effect on the NH_4_^+^ concentration in rhizosphere soil (Figure 5a). There were no significant differences in the nitrate nitrogen (NO_3_^−^) concentrations under the PP-MPs and HDPE-MPs compared with CK, while there was a significant difference under the PLA-MPs (*p* < 0.05), and the increase was the highest, being 437.63% (Figure 5b). The total nitrogen (TN) concentration was not significantly different under the PP-MPs compared with CK, while the TN concentrations were significantly different under the HDPE-MPs and PLA-MPs (*p* < 0.05), and the reduction rates were 26.65% and 22.30%, respectively, which indicated that the HDPE-MPs and PLA-MPs had significant effects on the reductions in the TN concentrations in rhizosphere soil (Figure 5c). There were significant differences in the total phosphorus (TP) concentrations under different microplastic types compared with CK (*p* < 0.05), and the TP concentrations were increased by 15.32%, 29.08%, and 16.88% under the PP-MPs, HDPE-MPs, and PLA-MPs, respectively; the increase in the TP concentration was the largest under the HDPE-MPs, indicating that the HDPE-MPs had the greatest effect on the TP concentration in rhizosphere soil (Figure 5d). The pH values showed slightly decreasing trends under the PP-MPs, HDPE-MPs, and PLA-MPs compared with CK, while there was a significant difference in the pH value under the PLA-MPs with a decrease of 2.18% (*p* < 0.05) (Figure 5e). The differences in electrical conductivity (EC) were not significant under the PP-MPs, HDPE-MPs, and PLA-MPs compared with CK, and the EC had a decreasing trend with a reduction rate of 10.09% under the PP-MPs, while the EC was increased by 5.10% and 10.38% under the HDPE-MPs and PLA-MPs, respectively (Figure 5f). The differences in the OM concentrations were not significant under the PP-MPs, HDPE-MPs, and PLA-MPs compared with CK, and the decrease in the OM concentration under the HDPE-MPs was small, while the OM concentrations were increased by 1.80% and 6.41% under the PP-MPs and PLA-MPs, respectively, indicating that the PLA-MPs had the greatest effect on the OM concentration in rhizosphere soil (Figure 5g).

As shown in Figure 6 and Table 3, there were significant differences in the NH_4_^+^ concentrations under different microplastic particle sizes compared with CK in rhizosphere soil (*p* < 0.05), and the NH_4_^+^ concentrations increased by 66.96%, 54.48%, and 48.30% under different particle sizes (150, 1000, and 4000 μm); the increase in the NH_4_^+^ concentration was the largest under the low particle size (150 μm), indicating that the increase in NH_4_^+^ concentrations decreased with the increase in microplastic particle sizes, and the effect on the increase in the NH_4_^+^ concentration was the largest under the low particle size (150 μm) (Figure 6a). There was no significant difference in the NO_3_^−^ concentration under the low particle size (150 μm) compared with CK, but there were significant differences in the NO_3_^−^ concentrations under the medium particle size (1000 μm) and high particle size (4000 μm) (*p* < 0.05), and the increases were 241.74% and 297.50%, respectively (Figure 6b). The TN and TP concentrations had significant differences under different particle sizes compared with CK (*p* < 0.05), and the TN and TP concentrations decreased by 22.01% and 18.46%, 14.68% and 23.47%, and 18.53% and 19.34% under different particle sizes (150, 1000, and 4000 μm), respectively; the reduction in the TN concentration and the increase in the TP concentration had the greatest effect under the low particle size (150 μm) and medium particle size (1000 μm), respectively (Figure 6c,d). The pH values showed slightly decreasing trends under different particle sizes compared with CK, but the differences were not significant; the pH values decreased by 1.33% and 1.51% under the medium particle size (1000 μm) and high particle size (4000 μm), respectively (Figure 6e). The EC had no significant differences under different particle sizes compared with CK, while the EC had a decreasing trend under the low particle size (150 μm), and the reduction rate was 2.41%; however, the EC increased by 2.72% and 5.07% under the medium particle size (1000 μm) and high particle size (4000 μm), respectively (Figure 6f). The OM concentrations were not significantly different under different particle sizes compared with CK, and the decrease in the OM concentration was small under the high particle size (4000 μm), while the OM concentrations increased by 3.45% and 4.34% under the low particle size (150 μm) and medium particle size (1000 μm), respectively, indicating that the increase in the OM concentration had the greatest effect under the medium particle size (1000 μm) in rhizosphere soil (Figure 6g).

As shown in Figure 7 and Table 3, there were significant differences in the NH_4_^+^ and TP concentrations under different microplastic concentrations compared with CK (*p* < 0.05), and the NH_4_^+^ and TP concentrations increased by 68.84% and 19.13%, 51.22% and 20.64%, and 49.67% and 21.51% under each microplastic concentration (0.1, 0.5, and 1 g·kg^−1^), respectively; the increase in the NH_4_^+^ concentration decreased but the increase in the TP concentration increased with the increase in microplastic concentrations, and the increase in the NH_4_^+^ and TP concentrations had the greatest effect under the low concentration (0.1 g·kg^−1^) and high concentration (1 g·kg^−1^), respectively (Figure 7a,d). The NO_3_^−^ concentrations had no significant difference under different microplastic concentrations compared with CK; however, the NO_3_^−^ concentrations were relatively high under each microplastic concentration (0.1, 0.5, and 1 g·kg^−1^), and the increases were 195.14%, 168.25%, and 201.86%, respectively, indicating that the NO_3_^−^ concentration had the greatest effect under the high concentration (1 g·kg^−1^) in rhizosphere soil (Figure 7b). The TN concentrations had significant differences under different concentrations compared with CK (*p* < 0.05), and the TN concentrations decreased by 18.01%, 19.42%, and 17.79% under each microplastic concentration (0.1, 0.5, and 1 g·kg^−1^), respectively; the reduction in the TN concentration had the greatest effect under the medium concentration (0.5 g·kg^−1^) in rhizosphere soil (Figure 7c). There were no significant differences in the pH values under the low concentration (0.1 g·kg^−1^) and medium concentration (0.5 g·kg^−1^) compared with CK, but there was a significant difference (*p* < 0.05) and the pH value decreased by 1.27% under the high concentration (1 g·kg^−1^) (Figure 7e). The EC and OM concentrations had no significant differences under different microplastic concentrations compared with CK, and the increases in EC and OM concentrations under each microplastic concentration (0.1, 0.5, and 1 g·kg^−1^) were 3.57% and 1.68%, 1.28% and 2.28%, and 0.54% and 3.35% respectively; the increase in the EC decreased but the increase in the OM concentration increased with the increase in microplastic concentrations, and the increase in the EC and OM concentrations had the greatest effect under the low concentration (0.1 g·kg^−1^) and high concentration (1 g·kg^−1^) in rhizosphere soil (Figure 7f,g).

### 2.5. Effects of MPs on Enzymatic Activities of Wheat Rhizosphere Soil

**Figure 8:**
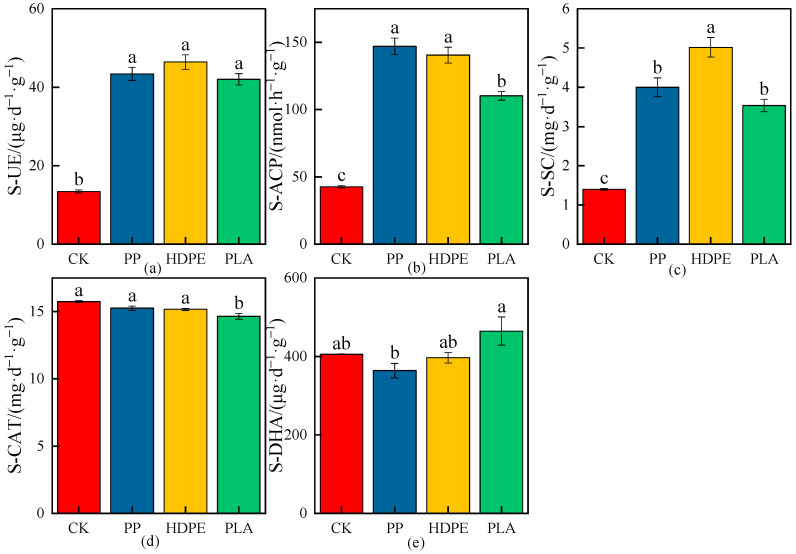
Effects on enzymatic activities of wheat rhizosphere soil under the CK and different microplastic types. (**a**) S-UE, (**b**) S-ACP, (**c**) S-SC, (**d**) S-CAT, and (**e**) S-DHA. All data are presented as the mean ± SD (n ≥ 3 biological replicates). Lowercase letters above bars indicate significant differences according to the post hoc Fisher LSD test (*p* < 0.05).

**Figure 9:**
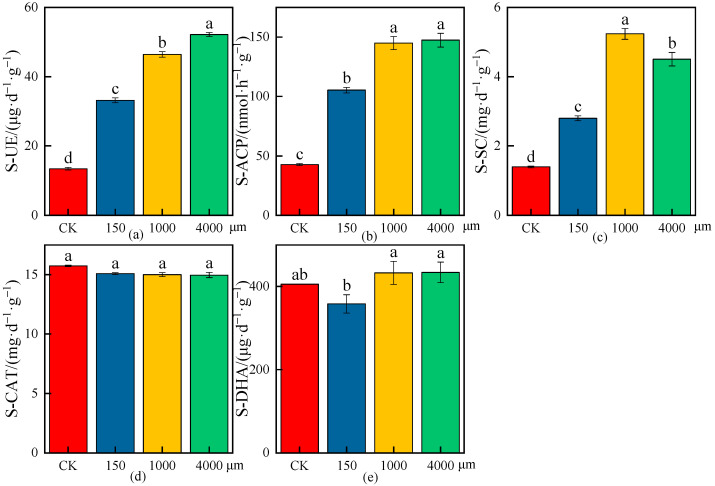
Effects on enzymatic activities of wheat rhizosphere soil under the CK and different microplastic particle sizes. (**a**) S-UE, (**b**) S-ACP, (**c**) S-SC, (**d**) S-CAT, and (**e**) S-DHA. All data are presented as the mean ± SD (n ≥ 3 biological replicates). Lowercase letters above bars indicate significant differences according to the post hoc Fisher LSD test (*p* < 0.05).

**Figure 10:**
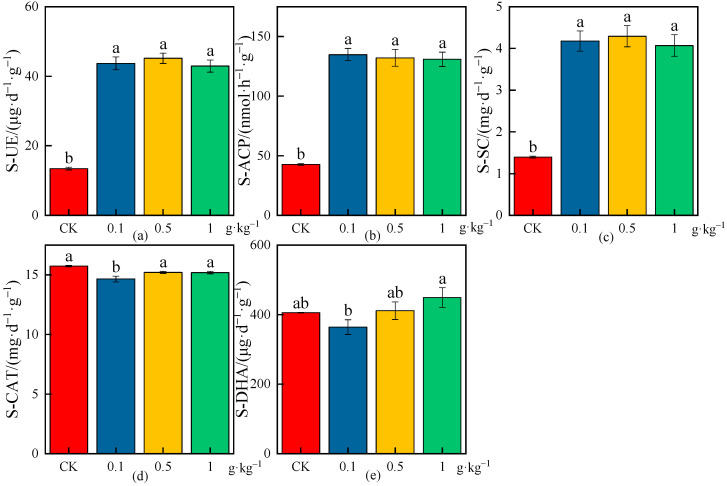
Effects on enzymatic activities of wheat rhizosphere soil under the CK and different microplastic concentrations. (**a**) S-UE, (**b**) S-ACP, (**c**) S-SC, (**d**) S-CAT, and (**e**) S-DHA. All data are presented as the mean ± SD (n ≥ 3 biological replicates). Lowercase letters above bars indicate significant differences according to the post hoc Fisher LSD test (*p* < 0.05).

The effects of different MPs on the average values of enzymatic activities in rhizosphere soil are shown in Figure 8, Figure 9 and Figure 10, and the increase or decrease ranges of the average values of enzymatic activities in rhizosphere soil under various treatments compared with CK are shown in Table 4.

As shown in Figure 8 and Table 4, the soil urease (S-UE) and soil sucrase (S-SC) activities had significant differences under different microplastic types compared with CK (*p* < 0.05), and the S-UE and S-SC activities increased by 222.85% and 186.00%, 245.23% and 259.18%, and 212.58% and 152.81% under the PP-MPs, HDPE-MPs, and PLA-MPs, respectively; the S-UE and S-SC activities increased most under the HDPE-MPs, indicating that the S-UE and S-SC activities had the greatest effect under HDPE-MPs in rhizosphere soil (Figure 8a,c). The soil acid phosphatase (S-ACP) activities had significant differences under different microplastic types compared with CK (*p* < 0.05) and increased by 243.69%, 228.54%, and 157.59% under the PP-MPs, HDPE-MPs, and PLA-MPs, respectively; the S-ACP activity increased the most under the PP-MPs, indicating that the S-ACP activity had the greatest effect under PP-MPs in rhizosphere soil (Figure 8b). There were no significant differences in the soil catalase (S-CAT) activities under the PP-MPs and HDPE-MPs compared with CK, while there was a significant difference in the S-CAT activity under the PLA-MPs (*p* < 0.05), and the maximum reduction was 6.98%, which indicated that the reduction in S-CAT activity had the greatest effect under the PLA-MPs in rhizosphere soil (Figure 8d). The soil dehydrogenase (S-DHA) activities had no significant differences under different microplastic types compared with CK, and the decrease in S-DHA activity was the largest, 10.25%, under the PP-MPs, while the increase in S-DHA activity was the highest, 14.55%, under the PLA-MPs (Figure 8e).

As shown in Figure 9 and Table 4, the S-UE and S-ACP activities had significant differences under different microplastic particle sizes compared with CK (*p* < 0.05), and they increased by 146.86% and 146.41%, 245.55% and 238.88%, and 288.26% and 244.53% under the different particle size (150, 1000, and 4000 μm), respectively; the S-UE and S-ACP activities increased the most under the high particle size (4000 μm), indicating that the S-UE and S-ACP activities increased with the increase in microplastic particle sizes, and the S-UE and S-ACP activities had the greatest effect under the high particle size (4000 μm) in rhizosphere soil (Figure 9a,b). The S-SC activities had significant differences under each particle size compared with CK (*p* < 0.05) and were increased by 100.60%, 274.82%, and 222.58% under different particle sizes (150, 1000, and 4000 μm), respectively; the S-SC activity increased the most under the medium particle size (1000 μm), indicating that the increase in S-SC activity had the greatest effect under the medium particle size (1000 μm) in rhizosphere soil (Figure 9c). There were no significant differences in the S-CAT activities under the different particle sizes compared with CK, but the S-CAT activities showed slight decreasing trends under different particle sizes (150, 1000, and 4000 μm) (Figure 9d). There were no significant differences in the S-DHA activities under different particle sizes compared with CK, and the S-DHA activities increased under the medium particle size (1000 μm) and high particle size (4000 μm), while the decrease in S-DHA activity was the largest, 11.64%, under the low particle size (150 μm), which indicated that the reduction in the S-CAT activity had the greatest effect under the lower particle size (150 μm) in rhizosphere soil (Figure 9e).

As shown in Figure 10 and Table 4, there were significant differences in the S-UE and S-SC activities under different microplastic concentrations compared with CK (*p* < 0.05), and they were increased by 224.94% and 199.20%, 236.12% and 207.40%, and 219.61% and 191.40% under each microplastic concentration (0.1, 0.5, and 1 g·kg^−1^), respectively; the S-UE and S-SC activities increased the most under the medium concentration (0.5 g·kg^−1^), indicating that the increase in S-UE and S-SC activities had the greatest effect under the medium concentration (0.5 g·kg^−1^) in the rhizosphere soil (Figure 10a,c). The S-ACP activities had significant differences under different microplastic concentrations compared with CK (*p* < 0.05) and were increased by 215.09%, 208.74%, and 205.99% under the different microplastic concentrations (0.1, 0.5, and 1 g·kg^−1^), respectively, which indicated that the increase in S-ACP activity decreased with the increase in microplastic concentrations, and the increase in S-ACP activity had the greatest effect under the low concentration (0.1 g·kg^−1^) in rhizosphere soil (Figure 10b). The S-CAT activity had a significant difference under the low concentration (0.1 g·kg^−1^) compared with CK (*p* < 0.05), and the maximum reduction was 6.90%; there were no significant differences in the S-CAT activities under the medium concentration (0.5 g·kg^−1^) and high concentration (1 g·kg^−1^) (Figure 10d). There were no significant differences in the S-DHA activities under different particle sizes compared with CK, and the decrease in S-DHA activity was the highest, 10.14%, under the low concentration (0.1 g·kg^−1^), indicating that the treatment had the greatest effect on the decrease in S-DHA activity; the increase in S-DHA activity was the highest at 10.76% under high-concentration (0.1 g·kg^−1^) treatment, indicating that the treatment had the greatest effect on the increase in S-DHA activity in rhizosphere soil (Figure 10e).

## 3. Discussion

### 3.1. Effects of Different MPs on Wheat Growth

Some studies have been conducted on the effects of MPs on wheat growth; the relevant studies showed that the *GR* of wheat seeds was inhibited under low/medium concentrations (<500 mg·L^−1^), and the *GIR* ranged from 2.86% to 20%, while the *GR* of wheat seeds was promoted under the high concentration (1000 mg·L^−1^) compared with the CK; the inhibitory effect on *VI* was strongest for LDPE-MPs, followed by EVA-MPs and then PMMA-MPs, and the promotion of the EVA-MPs and LDPE-MPs at the low concentration was higher than that at the high concentration, and the growth characteristics of wheat seeds had no significant effect under PMMA-MPs [44]. The PS-MPs and PVC-MPs could promote the *GR* of wheat seeds under a low concentration and the *GIR* under medium/high concentrations; the PVC-MPs could promote the *GI*, *GP*, and *MGT* of wheat seeds, and PS-MPs could promote the biomass of wheat [45]. There was a significant inhibition of the elongation of wheat roots and stems under the high concentration (200 mg·L^−1^) of the PS-MPs, and the inhibition rates were 67.15% and 56.45%, respectively [46]; the growth parameters and chlorophyll concentration of wheat had a significant increasing trend under PS-MPs [47]. The effects of PE-MPs on the *GR* of wheat seed were characterized by “low inhibition, medium promotion, and high inhibition”, while the effects of PP-MPs were characterized by “low promotion, high inhibition” [48]. Chen et al. found that the basic growth indicators of wheat (plant height, leaf area, leaf fresh weight, and root fresh weight) had certain negative effects under different particle sizes and concentrations of PP-MPs, and the inhibitory effects were more obvious under small-size PP-MPs [49]. From a mechanistic perspective, MPs can easily clog the soil pores and reduce the flow of water and nutrients, and MPs are highly hydrophobic and easily adhere to the surface of roots when they enter the soil, blocking the pores of roots, preventing plants from absorbing water and nutrients, and inhibiting the growth of crop roots. In addition, the water absorption ability is inhibited when the crops are subjected to external MP stress, and in order to absorb deep water, the crops themselves regulate the root growth ability. However, if the inhibition effect exceeds the water stress resistance of plants, plant growth and development will be significantly inhibited.

The above research was basically conducted by indoor hydroponics and soil culture, while this study was carried out under natural environmental conditions by pot tests, so the research results were closer to the actual situation of agricultural production. Overall, this study showed that the effects of MPs on the average *GIR* of wheat seeds were in the order of HDPE-MPs > PLA-MPs > PP-MPs, and the inhibitory effects on the growth of wheat seeds were in the order of PLA-MPs > HDPE-MPs > PP-MPs, indicating that the inhibitory effect of the PLA-MPs increased with the growth of wheat seeds, which might be due to the fact that the PLA-MPs was degraded with the increase in time as PLA is biodegradable, which increased the effect on the growth of wheat seeds, while the PP-MPs had better stability in soil. The effects of the medium particle size or medium concentration of MPs on wheat seed growth characteristics were the most significant, while the effects of low/high particle sizes and concentrations were weakened, which might be caused by the characteristics of MPs themselves or the agglomeration of MPs reducing the accessibility of wheat to MPs; the mechanism of the effect of MPs on plants is still unclear.

### 3.2. Effects of MPs on Soil Physicochemical Properties and Enzymatic Activities

MPs have a wide range of impacts on soil ecological and environmental systems, mainly including physicochemical properties and biogeochemical cycles [50,51,52]. Wang et al. [36] showed that the pH value had an increasing trend under PBAT/PLA-MPs, and LDPE-MPs significantly increased the pH value [53]; Liu et al. [39] and Janczak et al. [54] found that PLA-MPs significantly reduced the pH value, and Huang et al. [55] showed that PE-MP residual film significantly reduced the pH value during the whole growth period of pepper. In addition, some studies have also pointed out that the addition of LDPE-MPs had little effect on the pH value but could significantly improve soil cation exchange capacity [56]; this study showed that different MPs all reduced the pH value of wheat rhizosphere soil, and the pH value decreased by 2.18% under the PLA-MPs. The accumulation of MPs will affect the nitrogen and phosphorus cycling process in the soil ecosystem and has different effects under different microplastic conditions [57]. For example, the NO_3_^−^ and available phosphorus had significant decreasing trends under LDPE-MPs [56], PS-MPs significantly reduced the available phosphorus and NO_3_^−^ [58], PLA-MPs and PE-MPs significantly reduced the NH_4_^+^ and NO_3_^−^ concentrations [59], and PE-MPs significantly increased the NH_4_^+^ concentration [60]; Li et al. [31] showed that PP-MPs promoted an increase in the TP concentration, while PBAT-MPs had no significant effect on TP. The relevant studies indicated that the addition of the PE-MPs would promote the conversion of NH_4_^+^ into NO_3_^−^ [61], and the NH_4_^+^ and NO_3_^−^ concentrations with MPs added would constantly change during the experiment [62]; this study showed that MPs significantly increased NH_4_^+^ and TP in wheat rhizosphere soil, while significantly decreasing TN.

Relevant studies showed that the EC had a significantly decreasing trend under PE-MPs [55], while this study found that the EC had a certain increasing but non-significant trend under different MPs in wheat rhizosphere soil. PBAT/PLA-MPs significantly increased the OM concentration, while PE-MPs had no significant effect on the OM concentration [63]; Yu et al. [64] found that the addition of PE-MPs increased the OM concentration, Lu et al. [65] found that biodegradable white film increased the OM concentration by 2.93 g·kg^−1^, which was consistent with the increase in the OM concentration caused by the addition of MPs in this study. Considering the enzymatic activities under MPs in soil, relevant studies showed that S-CAT and soil alkaline phosphatase (S-AKP) activities had significant increasing trends under different mass fractions of MPs compared with CK, while S-SC activity had a significant decreasing trend under 5% treatment [60]; PE-MPs could promote S-DHA and S-UE activities and inhibit S-SC activity [66]; the addition of LDPE-MPs promoted an increase in S-UE and FDAse activities but inhibited S-AKP activity [35]; the addition of PE-MPs increased S-AKP activity and inhibited the activity of S-UE, an enzyme related to the N cycle [67]; this study showed that S-UE, S-ACP, and S-SC activities had significant increasing trends under different MPs, while S-CAT activities had significant decreasing under PLA-MPs and the low concentration (0.1 g·kg^−1^).

Overall, plastic materials will gradually decompose and form MPs with different particle sizes in the soil environment, and the physicochemical properties will be altered in the soil. For example, MPs can increase the evaporation rate of soil moisture as a pathway for soil moisture transport and can tightly bind with soil aggregates, thereby affecting the bulk density, water-holding capacity, and particle size classification of water-stable aggregates in soil. In addition, MPs can enhance the adsorption and reactivity of soil, and reactivity changes the pH, salinity, OM, nutrient elements, and ion states. Therefore, MPs indirectly have adverse effects on plant growth while affecting soil water cycling and nutrient transport. MPs also can provide certain adsorption sites for soil microorganisms, and the accumulation of bacteria on their surface will have higher biological toxicity, thus reducing the amount, activity, and diversity of soil microorganisms, damaging the soil microbial environment and affecting the soil ecological environment required for plant growth.

## 4. Materials and Methods

### 4.1. Test Materials

#### 4.1.1. Test Soil and Treatment

In August 2020, test soil was collected from the sandy soil (0~20 cm) in the farmland layer around the Agricultural Water Soil Environmental Field Research Station of Xinxiang, Chinese Academy of Agricultural Sciences. The soil was naturally air-dried to screen out the visible animal and plant residues, stones, etc., and then the soil was screened with a 5 mm screen for use. The basic physicochemical properties of the tested soil and the abundance of MPs were as follows: the main composition of soil particle sizes was 6.85% less than 0.002 mm, 52.61% from 0.002 to 0.02 mm, and 40.54% from 0.02 to 2.0 mm; the texture of the soil tested was sandy loam, the dry volume mass was 1.45 g·cm^−1^, pH value was 7.89, the TN concentration was 0.752 g·kg^−1^, the TP concentration was 0.603 g·kg^−1^, the OM concentration was 24.14 g·kg^−1^, the EC was 163.90 μS·cm^−1^, the NH_4_^+^ concentration was 0.003 g·kg^−1^, the NO_3_^−^ concentration was 0.294 g·kg^−1^, and the abundance of MPs was 12 n·kg^−1^. The soil particle size was determined by laser particle size analyzer, the dry volume mass was measured by the cutting ring method, the pH value was determined by a glass electrode pH meter (sod–water ratio 1:2.5), the concentrations of NH_4_^+^, NO_3_^−^, TN, and TP were determined by a continuous-flow analyzer (Auto Analyzer 3 produced by BRAN + LUEBBE, Hamburg, Germany), EC was determined by a conductivity meter, OM was determined by potassium dichromate oxidative external heating method, and the abundance of MPs was identified by an Agilent 8700 laser direct infrared (Agilent 8700 produced by Agilent Technologies Inc., Santa Clara, CA, USA; LDIR) chemical imaging system; refer to the references for the specific steps [68,69,70]. 

#### 4.1.2. Test Seeds and Treatment

Bainong 4199 is a gramineous wheat plant, and it passed the national wheat audit of China (No. 20210049) in 2021. According to the certification announcement, the variety of Bainong 4199 is semi-winter, the whole growth period is 226.5 days, the plant height is about 71.5 cm, the thousand-grain weight is 44.1 g, the grain bulk weights are 799 g/L and 787 g/L, the protein concentrations are 14.7% and 13.5%, the wet gluten concentrations are 33.6% and 28.7%, the stability times are 6.1 min and 8.7 min, the water absorptions are 58% and 56%, and the stretch area is 81 cm^2^. Bainong 4199 is suitable for planting in some areas of Henan, Shaanxi, Jiangsu, and Anhui provinces. Before the experiment, wheat seeds with full grain and a healthy state were selected, and the seeds were mixed with phoxius (an organophosphate insecticide) and the surface layer of wheat seeds to prevent the wheat seeds from being destroyed in the soil.

#### 4.1.3. Selection and Treatment of MPs

To analyze the effects of biodegradable and non-biodegradable MPs on wheat’s growth and rhizosphere soil environment, three microplastic types (PP-MPs, HDPE-MPs, and PLA-MPs), particle sizes (150, 1000, and 4000 μm), and concentrations (0.1, 0.5, and 1 g·kg^−1^) were selected. The MPs were purchased from Dongguan Xinli Plastic Raw Materials Co., Ltd. (Dongguan, Guangdong, China), and manufactured according to the particle size requirements, and the concentration of MPs was mainly performed according to the test requirements in accordance with a certain ratio of MP quality and soil quality. PP is a semi-crystalline thermoplastic polymer made from propylene monomer by addition polymerization, with a density of 0.89~0.92 g/cm^3^ and a melting point of 164~176 °C; HDPE is an opaque white wax material with a density of 0.94~0.95 g/cm^3^ and a melting point of 125~135 °C; PLA is a new biodegradable material with a density of 1.25~1.28 g/cm^3^ and a melting point of 176 °C.

### 4.2. Test Design and Process

**Figure 11:**
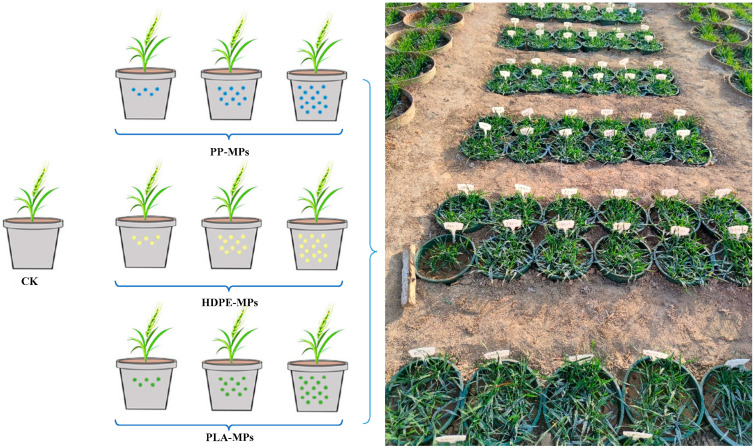
Concept and actual image of pot experiment design.

The experiment was carried out in the natural environment of the Agricultural Water Soil Environmental Field Research Station of Xinxiang, Chinese Academy of Agricultural Sciences, China. The control group (CK) without MPs was set up, with a total of 28 treatments, each of which was repeated three times (Figure 11). For each treatment, 5 kg of test soil was used; different MPs were added to the soil, fully and evenly mixed, and the soil was placed in plastic flower pots. The wheat seeds were sown in the treated flower pots on 25 October 2020, and 25 wheat seeds were sown in each pot. The number of germinated wheat seeds per day was recorded within 7 days after sowing. The SPAD and nitrogen concentration of the leaves were measured by a chlorophyll analyzer (TY-4N) in December 2020, February 2021, and May 2021 respectively, and the leaves of the same size were selected for each measurement. The plant height of wheat was measured by a centimeter ruler before harvest in June 2021, and 10 fixed wheat plants were measured in each pot; the SPAD, nitrogen concentration of the leaves, and plant height under each treatment were the average values of 30 wheat plants measured.

### 4.3. Measurement Index Method

#### 4.3.1. Growth Index Measurement Method

In order to analyze the effects of different MPs on wheat germination, the *GR*, *GP*, *GI*, *VI*, *MGT*, and *GIR* of the wheat were calculated according to the experimental data and the following related formulas [29,44,71]:

(1) $$GR=\frac{\mathrm{Number}\;\mathrm{of}\;\mathrm{germination}\;\mathrm{of}\;\mathrm{wheat}\;\mathrm{seeds}\;\mathrm{in}\;7\;\mathrm{days}}{\mathrm{Total}\;\mathrm{seed}\;\mathrm{sown}}\times 100\%$$

(2) $$GP=\frac{\mathrm{Number}\;\mathrm{of}\;\mathrm{germination}\;\mathrm{of}\;\mathrm{wheat}\;\mathrm{seeds}\;\mathrm{in}\;3\;\mathrm{days}}{\mathrm{Total}\;\mathrm{seed}\;\mathrm{sown}}\times 100\%$$

(3) $$GI=\sum \left(G_{t}/D_{t}\right)$$

(4) VI=GI×S

(5) MGT=∑(F×X)/∑F

(6) $$GIR=\frac{\mathrm{Number}\;\mathrm{of}\;\mathrm{germination}\;\mathrm{in}\;\mathrm{CK}-\mathrm{Number}\;\mathrm{of}\;\mathrm{germination}\;\mathrm{in}\;\mathrm{treatment}\;\mathrm{group}}{\mathrm{Number}\;\mathrm{of}\;\mathrm{germination}\;\mathrm{in}\;\mathrm{treatment}\;\mathrm{group}}\times 100\%$$

where *G_t_* is the number of sprouting days on the *t* day after the beginning of wheat germination, *D_t_* is the corresponding number of germination days, *S* is the seed length within a certain period, *F* is the number of new sprouting days of wheat seeds on the *X* day, and *X* is the number of germination days.

#### 4.3.2. Methods for the Determination of Soil Physicochemical Properties and Enzymatic Activity Indices

In this study, the physicochemical properties were mainly determined by the pH values, NH_4_^+^, NO_3_^−^, TN, TP, EC, and OM in wheat rhizosphere soil, and the test methods of physicochemical properties are shown in Section 4.1.1. The main indices of enzymatic activities were S-UE, S-ACP, S-SC, S-DHA, and S-CAT activities, and the indophenol blue colorimetric method, *p*-nitrophenol colorimetric method, DNS colorimetric method, TTC reduction method, and UV colorimetric method were used for detection [72,73].

### 4.4. Data Processing

SPSS21.0 and Origin 2021 were used to conduct statistical analysis and analysis of variance on the experimental data and plot them. In the analysis of variance, a 95% confidence level was selected, and the Fisher LSD test was applied to conduct multiple comparative analysis of the differences between different treatments.

## 5. Conclusions

The results showed that the different types, particle sizes, and concentrations of MPs had different effects on wheat seed germination and growth characteristics, plant growth characteristics, physicochemical properties, and enzyme activities in the rhizosphere soil. Our work confirmed the greatest inhibitory effect on wheat seed germination occurred under the HDPE-MPs, medium particle size, and medium concentration of MPs, and the wheat seed and plant growth characteristics had significant decreasing trends under the PLA-MPs. The decreases in SPAD and the nitrogen concentration of the leaves were the largest under the low particle size and low concentration of MPs, while the decreases in plant height were the largest under the high particle size and high concentration of MPs. NH_4_^+^, TP, S-UE, S-ACP, and S-SC had significant increasing trends under different MPs, while TN, pH, and S-CAT had significant decreasing trends under the PLA-MPs. The results of this study can provide data support and technical support for the study of the mechanism by which MPs influence crop growth and the effects of rhizosphere soil ecological environments.

## Figures and Tables

**Table 1:** The increase or decrease ranges of wheat seed growth characteristics under various treatments compared with CK (%).

Treatments	*GP*	*GI*	*VI*	*MGT*
PP-MPs	−16.42	−7.13	−23.33	1.73
HDPE-MPs	−46.25	−17.01	−40.49	3.78
PLA-MPs	−66.55	−21.00	−55.98	4.67
150 μm	−35.26	−14.64	−39.65	3.05
1000 μm	−41.39	−16.52	−43.99	3.34
4000 μm	−43.56	−13.12	−32.78	3.83
0.1 g·kg^−1^	−40.12	−14.77	−34.21	3.52
0.5 g·kg^−1^	−49.04	−16.91	−42.63	4.23
1 g·kg^−1^	−31.83	−12.63	−39.37	2.46

**Table 2:** The increase or decrease ranges of SPAD, nitrogen concentration of the leaves, and plant height in wheat under various treatments compared with CK (%).

Treatments	SPAD	Nitrogen Concentration of Leaf	Plant Height
PP-MPs	−12.23	−8.85	−8.94
HDPE-MPs	−38.07	−33.12	−12.28
PLA-MPs	−53.74	−41.91	−20.77
150 μm	−34.31	−28.44	−10.47
1000 μm	−33.18	−27.96	−14.29
4000 μm	−29.82	−22.78	−16.77
0.1 g·kg^−1^	−38.04	−32.89	−12.46
0.5 g·kg^−1^	−34.04	−28.70	−12.92
1 g·kg^−1^	−25.74	−18.34	−16.04

**Table 3:** The increase or decrease ranges of the average values of physicochemical indices in rhizosphere soil under various treatments compared with CK (%).

Treatments	NH_4_^+^	NO_3_^−^	TN	TP	pH	EC	OM
PP-MPs	60.63	2.63	−6.28	15.32	−0.07	−10.09	1.80
HDPE-MPs	59.26	125.00	−26.65	29.08	−0.76	5.10	−0.90
PLA-MPs	49.84	437.63	−22.30	16.88	−2.18	10.38	6.41
150 μm	66.96	26.01	−22.01	18.46	−0.16	−2.41	3.45
1000 μm	54.48	241.74	−14.68	23.47	−1.33	2.72	4.34
4000 μm	48.30	297.50	−18.53	19.34	−1.51	5.07	−0.48
0.1 g·kg^−1^	68.84	195.14	−18.01	19.13	−0.85	3.57	1.68
0.5 g·kg^−1^	51.22	168.25	−19.42	20.64	−0.88	1.28	2.28
1 g·kg^−1^	49.67	201.86	−17.79	21.51	−1.27	0.54	3.35

**Table 4:** The increase or decrease ranges of the average values of enzymatic activities in rhizosphere soil under various treatments compared with CK (%).

Treatments	S-UE	S-ACP	S-SC	S-CAT	S-DHA
PP-MPs	222.85	243.69	186.00	−3.13	−10.25
HDPE-MPs	245.23	228.54	259.18	−3.65	−2.23
PLA-MPs	212.58	157.59	152.81	−6.98	14.55
150 μm	146.86	146.41	100.60	−4.12	−11.64
1000 μm	245.55	238.88	274.82	−4.69	6.70
4000 μm	288.26	244.53	222.58	−4.94	7.02
0.1 g·kg^−1^	224.94	215.09	199.20	−6.90	−10.14
0.5 g·kg^−1^	236.12	208.74	207.40	−3.41	1.46
1 g·kg^−1^	219.61	205.99	191.40	−3.45	10.76

## Data Availability

The original contributions presented in the study are included in the article; further inquiries can be directed to the corresponding authors.
